# Single Standard Substance for the Simultaneous Determination of Eleven Components in the Extract of Paeoniae Radix Alba (Root of *Paeonia lactiflora* Pall.)

**DOI:** 10.1155/2021/8860776

**Published:** 2021-05-15

**Authors:** Menglan Shen, Qiaoyan Zhang, Luping Qin, Binjun Yan

**Affiliations:** College of Pharmaceutical Science, Zhejiang Chinese Medical University, Hangzhou 310053, China

## Abstract

Paeoniae Radix Alba (PRA), an herbal drug produced from the root of *Paeonia lactiflora* Pall., is widely used in many herbal medicine prescriptions/preparations. Since the pharmacological effects of PRA come from multiple chemical components, it is important to establish a method for the determination of those components in PRA extracts with simple operation and low cost, which is more suitable to evaluate the quality of PRA extracts and optimize the extraction process. This work introduced the quantitative analysis of multicomponents with a single-marker (QAMS) method for the simultaneous determination of eleven bioactive components in PRA extracts, including gallic acid, oxypaeoniflorin, catechin, albiflorin, paeoniflorin, ethyl gallate, galloylpaeoniflorin, pentagalloylglucose, benzoic acid, benzoylpaeoniflorin, and paeonol. In the QAMS method established based on high performance liquid chromatography with diode array detection, only the reference substance of paeoniflorin was needed, and the other ten components were determined based on their relative correction factors (RCFs) to paeoniflorin. Moreover, the repeatability and robustness of the RCFs were studied with different column temperatures, detection wavelengths, flow rates, column types, and instruments. In method validation, good linearity (*r* > 0.999), stability, repeatability (RSD < 1.9%), and accuracy (recoveries within 96.1%–105.5%) were shown. Sample analyses showed that the QAMS method was consistent with the conventional external standard method. The established method provided a comprehensive, efficient, and low-cost tool for the routine quality evaluation of PRA extracts.

## 1. Introduction

Paeoniae Radix Alba (PRA) has a long history of medicinal use in China, which is produced from the root of *Paeonia lactiflora* Pall. (RPLP) by removing its root bark, boiling in water, and drying in the sunlight. PRA mainly contains components including monoterpene glycosides (*e.g.*, paeoniflorin, albiflorin, oxypaeoniflorin, benzoylpaeoniflorin, and galloylpaeoniflorin), tannins (*e.g.*, gallic acid, ethyl gallate, and pentagalloylglucose), paeonol, *etc.* [1, 2]. Modern pharmacological studies have shown that the components in PRA have analgesic [3, 4], hepatoprotective [5], and anti-inflammatory [6–8] effects. PRA is also used in many herbal medicine prescriptions/preparations, such as Shaoyao Gancao decoction, Danggui Shaoyao decoction, Shiquan Dabu pills, and Bazhen pills, *etc.* In the current Chinese Pharmacopoeia [9], PRA appears in 158 traditional Chinese medicine preparations, which ranks seven among all the traditional Chinese medicines. In the use of the prescriptions or the manufacturing of the preparations, PRA is usually extracted by water or aqueous ethanol first. The contents of the bioactive components extracted have a great influence on the quality of the final preparations, which determine their clinical effects. Therefore, a quantitative method for the bioactive components is needed to evaluate the quality of PRA extracts and optimize the extraction process.

The current Chinese Pharmacopoeia [9] only takes paeoniflorin as the quality-marker for content determination, which makes it difficult to comprehensively reflect the intrinsic quality of PRA, because it has been reported that the pharmacological effects of PRA come from not only paeoniflorin but also many other components [8]. In recent years, there are a variety of methods reported to analyze the components in PRA, such as HPLC [1], LC-MS [10], and NMR [11], *etc.* However, since some of the standard substances like oxypaeoniflorin, galloylpaeoniflorin, and benzoylpaeoniflorin are expensive as well as difficult to be prepared, the cost of the analysis will be relatively high, if multiple components are quantified using the conventional external standard method (ESM). Moreover, LC-MS and NMR methods are not so convenient as HPLC-UV method due to their high instrumental cost. Therefore, it is necessary to find a more low-cost and convenient method to evaluate the quality of PRA extracts. Quantitative analysis of multicomponents with a single-marker (QAMS) is a quantitative method based on the relative correction factors (RCFs) between the analytes, which uses only one reference substance instead of multiple reference substances [12, 13]. Since in varying analytical circumstances (*e.g.*, different instruments), the detection responses (correction factors) of different analytes usually change in the same direction (*i.e.*, increase or decrease simultaneously), the RCFs are almost unchanged and robust for quantification. QAMS method can be used to overcome the difficulties of reference substance shortage and high analytical cost [13–19]. However, in the reports on QAMS methods, the number of analytes was usually less than seven. With the increase in the number of analytes, the advantage of QAMS method will be more prominent.

**Figure 1:**
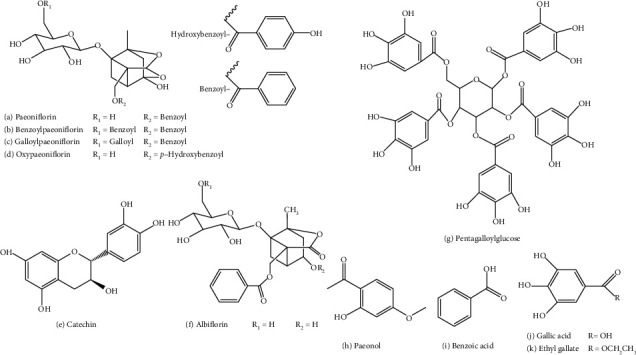
The structures of the eleven components determined. (a) Paeoniflorin. (b) Benzoylpaeoniflorin. (c) Galloylpaeoniflorin. (d) Oxypaeoniflorin. (e) Catechin. (f) Albiflorin. (g) Pentagalloylglucose. (h) Paeonol. (i) Benzoic acid. (j) Gallic acid. (k) Ethyl gallate.

For a better quality evaluation of PRA extracts with simple operation and low cost, this work developed an HPLC method to determine as many as eleven components, including gallic acid, oxypaeoniflorin, catechin, albiflorin, paeoniflorin, ethyl gallate, galloylpaeoniflorin, pentagalloylglucose, benzoic acid, benzoylpaeoniflorin, and paeonol (Figure 1), using only one reference substance of paeoniflorin, and investigated the feasibility and applicability of the QAMS method. Through a sufficient optimization of the separation conditions, the eleven components can be well separated simultaneously, allowing the use of UV detector rather than a more selective but expensive detector (like MS). Due to its low requirements on instruments and standard references, the proposed method is deemed to be convenient to use in future researches related to PRA and RPLP.

## 2. Materials and Methods

### 2.1. Materials

The RPLPs were collected from four different producing areas in China, including Baishan in Jilin province, Pan'an in Zhejiang province, Dongyang in Zhejiang province, and Hangzhou in Zhejiang province, and dried before use (*i.e.*, not processed by removing root bark and boiling in water like the prepared pieces of PRA). Prepared pieces of PRA were purchased from four different pharmacies in China, including Zhangzhongjing Pharmacy (Bozhou, Anhui, China), Henan Herbal Pharmacy (Bozhou, Anhui, China), Qixiangge (Jinhua, Zhejiang, China), and Renshoutang (Shaoxing, Zhejiang, China). The species identification was performed by Prof. Luping Qin (Zhejiang Chinese Medical University). The reference substance of gallic acid was purchased from Push Bio-Technology Co., Ltd. (Chengdu, Sichuan, China). The reference substances of oxypaeoniflorin, albiflorin, paeoniflorin, and benzoylpaeoniflorin were purchased from Victory Biological Technology Co., Ltd. (Chengdu, Sichuan, China). The reference substance of catechin was purchased from Shanghai Yuanye Bio-Technology Co., Ltd. (Shanghai, China). The reference substance of pentagalloylglucose was purchased from Xunchen Biological Technology Co., Ltd. (Chengdu, Sichuan, China). The reference substances of ethyl gallate and benzoic acid were purchased from Rhawn Science and Technology Co., Ltd. (Shanghai, China). The reference substance of paeonol was purchased from Jianglai Bio-Technology Co., Ltd. (Shanghai, China). Purities of all the reference substances determined by HPLC were more than 98%. HPLC grade acetonitrile and methanol were purchased from Tedia Co., Inc. (Fairfield, OH, USA). HPLC grade phosphoric acid was purchased from Tianjin Saifurui Technology Co., Ltd. (Tianjin, China). Deionized water was produced with a UPH-III-5T water purification system (Chengdu Chaochun Technology Co., Ltd., Chengdu, Sichuan, China).

### 2.2. Extraction of Paeoniae Radix Alba/Root of *Paeonia lactiflora* Pall

PRA/RPLP was pulverized to particle size below 0.85 mm. 5 g of the particles was extracted by 25 mL of 70% (v/v) ethanol with ultrasonic extraction method for 40 min. The extract was let to stand at room temperature and 70% (v/v) ethanol was added to make up the weight loss during the extraction. The extract was centrifuged at 16000 rpm for 5 min, and the supernatant was used for analysis.

### 2.3. HPLC Analyses

The HPLC analyses were carried out on a Waters e2695 HPLC system equipped with a 2998 photo diode array (PDA) detector (Waters Corp., Milford, MA, USA) using a Phenomenex Luna C18 column (5 *μ*m, 250 × 4.6 mm). Mobile phase A was acetonitrile and mobile phase B was 0.01% (v/v) phosphoric acid aqueous solution. Linear gradient elution was used as follows: 8% A in 0–20 min, 8–24% A in 20–50 min, 50% A in 50–60 min, 50–90% A in 60–68 min. The flow rate was 1.0 mL/min and the column temperature was 27°C. The injection volume was 10 *μ*L. The UV wavelengths were 274 nm for gallic acid, catechin, albiflorin, paeoniflorin, ethyl gallate, galloylpaeoniflorin, pentagalloylglucose, benzoic acid, benzoylpaeoniflorin, and paeonol and 257 nm for oxypaeoniflorin.

### 2.4. Preparation of Standard Solutions and Sample Solutions

The stock solutions of gallic acid, oxypaeoniflorin, catechin, albiflorin, paeoniflorin, ethyl gallate, galloylpaeoniflorin, pentagalloylglucose, and benzoylpaeoniflorin at concentrations of 4.975, 2.974, 0.7321, 7.051, 95.95, 12.62, 0.2959, 3.838, and 3.727 mg/mL were prepared, respectively, by dissolving each of the reference substances in 30% (v/v) methanol. The stock solutions of benzoic acid and paeonol at concentrations of 23.69 and 4.009 mg/mL were prepared, respectively, by dissolving each of the reference substances in methanol. According to the concentrations of the eleven components in the extracts, the mixed standard solutions were prepared by mixing appropriate amounts of the eleven stock solutions and diluting with 30% (v/v) methanol. The final concentrations of gallic acid, oxypaeoniflorin, catechin, albiflorin, paeoniflorin, ethyl gallate, galloylpaeoniflorin, pentagalloylglucose, benzoic acid, benzoylpaeoniflorin, and paeonol were 42.29, 108.5, 56.74, 409.0, 6141, 107.3, 136.0, 343.5, 236.9, 240.4, and 36.08 *μ*g/mL, respectively.

The PRA/RPLP extracts were directly analyzed as sample solutions without any sample pretreatments. All the samples and standard solutions were stored at 4°C and brought to room temperature before analysis.

### 2.5. Application of Relative Correction Factors for Quantitative Analysis of Multicomponents

In formula (1), *s* represents the internal standard (*i.e.*, paeoniflorin in this work) and *A*_*s*_ is the peak area acquired from *m*_*s*_ (the mass of component *s*). The correction factor (*f*_*s*_) is calculated as *m*_*s*_/*A*_*s*_. For component *x*, *A*_*x*_ is the peak area from *m*_*x*_ (the mass of component *x*), and the correction factor (*f*_*x*_) is calculated as *m*_*x*_/*A*_*x*_. The RCF of *x* to *s* (*f*_*x/s*_) can be calculated as *f*_*x*_/*f*_*s*_.

(1) $$\begin{array}{c} f_{x/s}=\frac{f_{x}}{f_{s}}=\frac{m_{x}/A_{x}}{m_{s}/A_{s}}. \end{array}$$

The RCF can be measured by HPLC analysis of the mixed standard solution. Then the content of component *x* (*m*_*x*_*'*) can be determined by HPLC analysis of the sample and calculated as formula (2).

(2) $$\begin{array}{c} m_{x}^{\prime }=f_{x/s}\frac{A_{x}^{\prime }}{A_{s}^{\prime }}m_{s}^{\prime }. \end{array}$$

*A*_*s*_′ and *A*_*x*_′ are the peak areas acquired from HPLC analysis of the sample solution. And *m*_*s*_′ is the content of paeoniflorin, which can be determined by conventional external standard method.

### 2.6. Method Validation

Following the International Conference on Harmonization guideline [20], the method validation was conducted, including linearity, limit of detection (LOD), limit of quantification (LOQ), precision, repeatability, stability, and accuracy.

## 3. Results and Discussion

### 3.1. Optimization of Analytical Conditions

The effect of acid added to the mobile phase on the separation was compared first, since the retention behavior of phenols and pentagalloylglucose in RP-HPLC columns can be significantly affected by the pH of the mobile phase [1, 21]. Oxypaeoniflorin/catechin and albiflorin/paeoniflorin could not be separated without an acidic mobile phase, and serious tailing peaks were observed. The addition of phosphoric acid in the mobile phase can reduce the peak widths of albiflorin and paeoniflorin to achieve better separation. When the concentration of phosphoric acid was 0.1% or 0.5% (v/v), poor separations of gallic acid, oxypaeoniflorin, and catechin were shown and tailing peaks of albiflorin and paeoniflorin were also observed. Then the concentration of phosphoric acid was reduced to 0.01% and resulted in better separation of these components.

Separation of the components with similar retention time (*e.g.*, oxypaeoniflorin/catechin) was a key point in the optimization of analytical conditions. After trying various gradient elution conditions, 8%A isocratic elution in 0–20 min and then gradient elution was better to separate the component pairs with close retention times, such as oxypaeoniflorin/catechin and galloylpaeoniflorin/pentagalloylglucose. Three different columns of AkzoNobel Kromasil C18 (5 *μ*m, 250 × 4.6 mm), Thermo Scientific ODS Hypersil C18 (5 *μ*m, 250 × 4.6 mm), and Phenomenex Luna C18 (5 *μ*m, 250 × 4.6 mm), different column temperatures (25, 27, 30, 35, and 40°C), and different flow rates (0.8, 1.0, and 1.2 mL/min) were investigated. In the Kromasil C18 column, the separation of oxypaeoniflorin and catechin was not good. The Hypersil C18 column was not able to separate gallic acid and oxypaeoniflorin from the interfering peaks near them. In contrast, the Luna C18 column was effective in the separation of oxypaeoniflorin, catechin, galloylpaeoniflorin, pentagalloylglucose, and benzoic acid. When the temperature was more than 30°C, gallic acid, oxypaeoniflorin, and catechin were not well separated. 27°C was chosen because the separations were better than those at 25°C. When the flow rate was 0.8 mL/min, gallic acid and galloylpaeoniflorin have poor separation and pentagalloylglucose could not be separated from benzoic acid. The flow rate of 1.0 mL/min could separate the components relatively well. Through a sufficient optimization of the separation conditions, the resolutions of the eleven components were all larger than 1.5.

**Figure 2:**
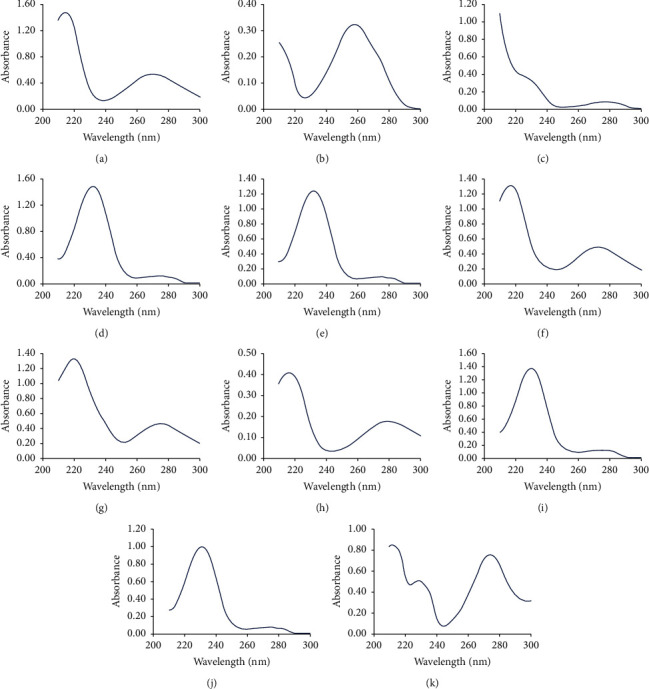
The UV spectra of the eleven components. (a) Gallic acid. (b) Oxypaeoniflorin. (c) Catechin. (d) Albiflorin. (e) Paeoniflorin. (f) Ethyl gallate. (g) Galloylpaeoniflorin. (h) Pentagalloylglucose. (i) Benzoic acid. (j) Benzoylpaeoniflorin. (k) Paeonol.

**Figure 3:**
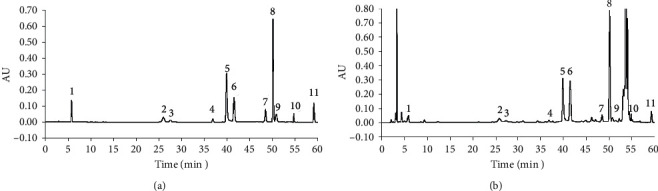
The chromatograms (274 nm) of (a) the mixed standard solution, (b) the sample solution. Peaks: 1. gallic acid; 2. oxypaeoniflorin; 3. catechin; 4. albiflorin; 5. paeoniflorin; 6. ethyl gallate; 7. galloylpaeoniflorin; 8. pentagalloylglucose; 9. benzoic acid; 10. benzoylpaeoniflorin; 11. paeonol.

The spectra of the eleven components acquired by the PDA detector are shown in Figure 2. Albiflorin, paeoniflorin, galloylpaeoniflorin, benzoylpaeoniflorin, and paeonol were determined at their maximum absorption wavelength (274 nm). The maximum absorption wavelengths of gallic acid, catechin, ethyl gallate, pentagalloylglucose, and benzoic acid were 270 nm, 278 nm, 271 nm, 280 nm, and 272 nm, respectively. These components were determined at the same wavelength of 274 nm because their spectra near 274 nm were all quite flat (Figure 2), and the sensitivities were similar to those at their maximum wavelengths. Oxypaeoniflorin was determined at its maximum absorption wavelength of 257 nm. The chromatograms acquired with the optimized analytical conditions are shown in Figure 3 (274 nm).

### 3.2. Method Validation

#### 3.2.1. Calibration Curves and Relative Correction Factors

Different volumes (2, 5, 10, 20, 35, and 50 *μ*L) of the mixed standard solution were injected into the instrument and analyzed. The linearity equations were calculated by least square regression method, taking the injected mass of the components as the *X* variables and the peak areas as the *Y* variables. All of the eleven components showed good linearity with correlation coefficients (*r*) all greater than 0.9994 (Table 1).

Paeoniflorin was selected as the internal standard because the retention time of paeoniflorin was in the middle and its concentration was usually the highest among the eleven components. The reference substance of paeoniflorin was relatively cheap, which was also a suitable factor for internal standard. The RCFs acquired from different injection volumes were close (Table 2) and the mean RCFs were used in the following work.

#### 3.2.2. Limits of Detection and Quantification

The mixed standard solutions were successively diluted with 30% (v/v) methanol into a series of standard solutions of different concentrations and analyzed. The LODs and the LOQs were measured at signal-to-noise ratios of 3 and 10, respectively. The results showed acceptable sensitivity of the method (Table 1).

#### 3.2.3. Precision

One extract was taken as the sample solution and repeatedly injected for 6 times to estimate the intraday precision. All the components showed good intraday precision with RSDs <2.0% (Table 3).

The intermediate precision was investigated by analyzing one sample solution on three days using two Waters HPLC systems. The RSDs of all the components were within 2.9% (Table 3).

To assess the repeatability, one extract was loaded into six sample vials and analyzed respectively, because there was no sample pretreatment conducted on the extract. The RSDs of all the components were within 2.0% (Table 3).

#### 3.2.4. Stability

The stability was assessed by analyzing one sample solution after being exposed at room temperature for 0, 1, 2, 4, 8, 12, and 24 h, respectively. As can be seen from the RSDs (Table 3), the sample was stable at room temperature within 24 h.

#### 3.2.5. Accuracy

The recovery experiments were conducted to measure the accuracy. The process was as follows: 0.1 mL of the mixed standard solutions of appropriate concentrations was spiked into 0.1 mL of the extracts (which were analyzed in advance), and the spiked samples were analyzed. The masses of the components in the extracts, the mixed standard solutions, and the spiked samples (Table 4) were calculated by multiplying their volumes (0.1 or 0.2 mL) by the concentrations determined. The recoveries of the eleven components were calculated, ranging from 96.1% to 105.5% (Table 4), which showed good accuracy.

### 3.3. Repeatability and Robustness of the Relative Correction Factors

To apply the QAMS successfully, good repeatability and robustness of the RCFs are the key issues. The repeatability of the RCFs was tested on three Waters HPLC systems in different laboratories. The results of mean RCFs and standard deviations were acquired from six different injection volumes (2, 5, 10, 20, 35, and 50 *μ*L) and listed in Table 5.

Some HPLC conditions including column temperatures, flow rates, type of columns, instruments, and detection wavelengths were slightly changed to evaluate the robustness of the RCFs (Table 6 and Table 7). By comparing the RCFs in Table 5 and Table 6, it can be seen that the RCFs were robust when the column temperature was biased within ±2 °C. When the flow rate was biased by −0.05 mL/min, −0.1 mL/min, and +0.02 mL/min, the RCFs were consistent as well. Because pentagalloylglucose and benzoic acid could not be well separated when the flow rate was biased by +0.05 mL/min or +0.1 mL/min, the RCF at the flow rate of 1.02 mL/min was measured instead. The YMC-Pack Pro C18 column (5 *μ*m, 250 × 4.6 mm) and the Waters BEH C18 column (5 *μ*m, 250 × 4.6 mm) could also be used to separate the components well, so they were used to measure the repeatability of the RCFs, and the results were close to those in the Phenomenex Luna C18 column. However, when these two columns were used, the flow rate was adjusted (Table 6) for a better separation of oxypaeoniflorin/catechin and pentagalloylglucose/benzoic acid. The RCFs measured on the Agilent 1260 HPLC system and the Dionex UltiMate 3000 HPLC system were close to those on the Waters HPLC systems.

The influences of biased detection wavelengths on the RCFs were evaluated by introducing ±1 and ± 2 nm of biases into the detection wavelengths (Table 7). For example, when there was -2 nm of bias, the peak areas of oxypaeoniflorin and paeoniflorin were acquired at 255 and 272 nm, respectively, which resulted in a *f*_oxypaeoniflorin/paeoniflorin_ of 0.0610. When the wavelengths were biased within ±2 nm, the RCFs (Table 7) were all close to the average RCFs in Table 5, except for catechin. The effects of biased detection wavelengths on the RCF of catechin were caused by its relatively large change of absorbance near 272 nm (Figure 2(c)).

### 3.4. Identification of the Peaks of the Analytes

When using QAMS, the identification of chromatographic peaks of the analytes can be achieved by combining the relative retention times (RRTs) and the UV spectra (Figure 2). The RRTs of the ten components to paeoniflorin in different columns and instruments were investigated (Table 8). The RRTs in the Phenomenex Luna C18 column and the YMC-Pack Pro C18 column were close. But the retention behaviors in Waters BEH C18 column were different from the other two columns. The elution order of pentagalloylglucose and benzoic acid was inversed in the YMC-Pack Pro column and the BEH column, compared to the Luna column. Therefore, when applying this method with different columns, it is necessary to identify the components according to their UV spectra (Figure 2). On the other hand, when using Agilent 1260 system and Dionex UltiMate 3000 system, the RRTs were nearly the same.

### 3.5. Comparison of the External Standard Method and the Quantitative Analysis of Multicomponents with a Single-Marker Method

To compare the QAMS method with the ESM, four batches of the RPLP and four batches of PRA were analyzed (Table 9). Deionized water and 70% (v/v) ethanol were used to extract these batches because they are two commonly used extracting solvents in the manufacturing of related herbal medicine preparations. The relative errors (REs) of the two methods were between −4.2% and +3.9%. The REs of oxypaeoniflorin in PRA were worse than other components because of the low contents of this component and the small peak areas. The contents of gallic acid were lower in the RPLPs extracted by 70% (v/v) ethanol compared with other batches. The contents of catechin in the 70% ethanol extracts of the RPLP from Pan'an and Dongyang were higher, close to the upper limit of the linear range (Table 1), which resulted in larger REs. The contents of albiflorin in the RPLPs from Pan'an, Dongyang, and Hangzhou were higher than that from Baishan. Ethyl gallate was only detected in the 70% ethanol extracts of the RPLP from Baishan and Hangzhou. Pentagalloylglucose could not be detected in the four batches of water extracts, possibly because of its low solubility in water. The contents of benzoic acid in PRA extracts were all lower than the LOD. Paeonol could not be detected in PRA and in the water extracts of RPLP.

## 4. Conclusions

In this work, a QAMS method based on HPLC was established, which took paeoniflorin as the reference substance and determined as many as eleven components simultaneously. Method validation showed that this method has good linearity, repeatability, stability, and accuracy. The proposed method provides an effective and low-cost tool for the quality assessment of PRA extracts. The method can be used to monitor and optimize the extraction process of PRA.

## Figures and Tables

**Table 1:** The calibration curves, LODs, and LOQs of the eleven components.

Component	Linearity equation	*r*	Linearity range (ng)	LOD (ng)	LOQ (ng)
Gallic acid	*Y* = 2939 *X *−* *7.236 × 10^4^	0.9999	84.57–2114	5.373	13.43
Oxypaeoniflorin	*Y* = 1714 *X *−* *1.486 × 10^5^	0.9999	217.1–5427	19.63	196.3
Catechin	*Y* = 713.1 *X *−* *3.237 × 10^4^	0.9999	113.5–2837	37.52	75.04
Albiflorin	*Y* = 93.82 *X *−* *1.700 × 10^4^	0.9999	818.0–2.045 × 10^4^	209.8	419.6
Paeoniflorin	*Y* = 103.0 *X *−* *3.256 × 10^5^	0.9999	1.228 × 10^4^–3.070 × 10^5^	344.7	689.4
Ethyl gallate	*Y* = 3158 *X *−* *1.939 × 10^5^	0.9999	214.6–5365	7.451	29.80
Galloylpaeoniflorin	*Y* = 1034 *X *−* *9.033 × 10^4^	0.9999	271.9–6798	20.27	40.53
Pentagalloylglucose	*Y* = 2380 *X *−* *5.623 × 10^4^	0.9994	687.0–1.718 × 10^4^	14.59	36.46
Benzoic acid	*Y* = 545.9 *X *−* *9.042 × 10^4^	0.9999	473.7–1.184 × 10^4^	41.02	82.04
Benzoylpaeoniflorin	*Y* = 171.2 *X *−* *2.513 × 10^4^	0.9999	480.8–1.202 × 10^4^	25.68	128.4
Paeonol	*Y* = 5077 *X *−* *9.662 × 10^4^	0.9999	72.16–1804	4.079	8.159

**Table 2:** The relative correction factors acquired with different injection volumes (2, 5, 10, 20, 35, and 50 *μ*L).

Relative correction factor	50 *μ*L	35 *μ*L	20 *μ*L	10 *μ*L	5 *μ*L	2 *μ*L	Mean	RSD (%)
*f*_gallic acid/paeoniflorin_	0.0350	0.0353	0.0354	0.0356	0.0353	0.0330	0.0349	2.8
*f*_oxypaeoniflorin/paeoniflorin_	0.0601	0.0613	0.0611	0.0637	0.0636	0.0592	0.0615	3.0
*f*_catechin/paeoniflorin_	0.146	0.144	0.148	0.153	0.155	0.148	0.149	2.9
*f*_albiflorin/paeoniflorin_	1.10	1.09	1.10	1.09	1.09	1.02	1.08	2.6
*f*_ethyl gallate/paeoniflorin_	0.0327	0.0327	0.0327	0.0329	0.0333	0.0326	0.0328	0.8
*f*_galloylpaeoniflorin/paeoniflorin_	0.0998	0.100	0.100	0.102	0.104	0.0992	0.101	1.7
*f*_pentagalloylglucose/paeoniflorin_	0.0436	0.0417	0.0412	0.0413	0.0418	0.0406	0.0417	2.4
*f*_benzoic acid/paeoniflorin_	0.189	0.189	0.195	0.194	0.193	0.181	0.190	2.6
*f*_benzoylpaeoniflorin/paeoniflorin_	0.603	0.603	0.607	0.613	0.615	0.604	0.607	0.9
*f*_paeonol/paeoniflorin_	0.0203	0.0203	0.0203	0.0204	0.0207	0.0193	0.0202	2.3

**Table 3:** The results of the precision, repeatability, and stability experiments.

Component	Intraday precision (RSD/%)	Intermediate precision (RSD/%)	Repeatability (RSD/%)	Stability (RSD/%)
Gallic acid	1.5	1.1	1.9	1.9
Oxypaeoniflorin	1.2	1.9	1.4	0.6
Catechin	0.6	1.7	1.7	0.5
Albiflorin	1.0	1.9	1.8	2.0
Paeoniflorin	0.9	2.2	1.9	2.0
Ethyl gallate	1.7	1.8	1.4	2.0
Galloylpaeoniflorin	0.8	1.8	1.9	1.8
Pentagalloylglucose	1.6	2.9	1.3	2.7
Benzoic acid	0.8	2.7	1.6	0.9
Benzoylpaeoniflorin	1.3	2.8	1.7	1.8
Paeonol	0.5	0.5	0.5	0.1

**Table 4:** The recoveries of the eleven components.

Component	Original (*μ*g)	Spiked (*μ*g)	Found (*μ*g)	Recovery (%)	Average recovery (%)	RSD (%)
Gallic acid	60.14	42.29	101.5	97.7	97.1	1.1
	58.34	42.29	100.1	98.7		
	51.15	42.29	92.43	97.6		
	53.03	42.29	93.75	96.3		
	53.94	42.29	94.60	96.1		
	31.25	42.29	72.02	96.4		

Oxypaeoniflorin	103.6	108.5	211.0	99.0	101.8	2.7
	103.8	108.5	212.1	99.8		
	82.36	108.5	195.9	104.6		
	88.36	108.5	200.9	103.7		
	88.18	108.5	201.6	104.5		
	114.2	108.5	222.1	99.4		

Catechin	32.29	56.74	91.44	104.3	105.5	1.9
	29.87	56.74	89.32	104.8		
	31.65	56.74	91.04	104.7		
	26.12	56.74	87.05	107.4		
	36.56	56.74	95.31	103.5		
	37.48	56.74	99.07	108.6		

Albiflorin	459.5	409.0	853.7	96.4	98.5	2.7
	440.2	409.0	858.7	102.3		
	367.3	409.0	758.7	95.7		
	385.3	409.0	772.1	94.6		
	308.5	409.0	723.7	101.5		
	365.0	409.0	776.5	100.6		

Paeoniflorin	7775	6141	13979	101.0	98.3	1.5
	7997	6141	13934	96.7		
	6275	6141	12336	98.7		
	6750	6141	12742	97.6		
	6720	6141	12741	98.1		
	6860	6141	12855	97.6		

Ethyl gallate	190.8	107.3	295.9	97.9	98.3	2.6
	186.0	107.3	296.7	103.2		
	162.2	107.3	268.1	98.7		
	159.2	107.3	262.3	96.0		
	166.5	107.3	270.2	96.6		
	216.8	107.3	321.5	97.5		

Galloylpaeoniflorin	167.1	136.0	306.8	102.8	104.6	0.9
	161.9	136.0	304.1	104.6		
	133.0	136.0	275.8	105.0		
	141.6	136.0	283.5	105.0		
	137.6	136.0	280.4	105.5		
	70.24	136.0	213.7	102.9		

Pentagalloylglucose	646.4	343.5	989.2	99.8	98.5	1.6
	643.5	343.5	977.8	97.3		
	525.3	343.5	873.0	101.2		
	583.6	343.5	919.4	97.8		
	571.1	343.5	905.4	97.3		
	570.9	343.5	906.3	97.6		

Benzoic acid	223.5	236.9	465.8	102.3	102.8	1.2
	226.2	236.9	475.1	105.1		
	199.7	236.9	442.8	102.6		
	218.3	236.9	461.5	102.7		
	202.0	236.9	445.5	102.8		
	125.6	236.9	365.4	101.2		

Benzoylpaeoniflorin	326.5	240.4	558.8	96.6	96.1	0.9
	333.1	240.4	561.7	95.1		
	272.8	240.4	505.7	96.9		
	302.2	240.4	533.2	96.1		
	273.6	240.4	502.0	95.0		
	297.9	240.4	531.0	97.0		

Paeonol	15.12	36.08	50.75	98.7	99.6	1.6
	13.40	36.08	49.08	98.9		
	14.50	36.08	51.52	102.6		
	14.52	36.08	50.67	100.2		
	17.93	36.08	53.49	98.6		
	25.16	36.08	60.84	98.9		

**Table 5:** The relative correction factors (mean ± standard deviation) acquired with three Waters HPLC systems.

Relative correction factor	Waters-1	Waters-2	Waters-3	Average
*f*_gallic acid/paeoniflorin_	0.0349 ± 0.0010	0.0351 ± 0.0008	0.0350 ± 0.0009	0.0350 ± 0.0009
*f*_oxypaeoniflorin/paeoniflorin_	0.0615 ± 0.0018	0.0623 ± 0.0017	0.0616 ± 0.0017	0.0618 ± 0.0018
*f*_catechin/paeoniflorin_	0.149 ± 0.004	0.151 ± 0.004	0.153 ± 0.004	0.151 ± 0.004
*f*_albiflorin/paeoniflorin_	1.08 ± 0.03	1.10 ± 0.01	1.10 ± 0.01	1.09 ± 0.0140
*f*_ethyl gallate/paeoniflorin_	0.0328 ± 0.0002	0.0330 ± 0.0004	0.0329 ± 0.0007	0.0329 ± 0.0005
*f*_galloylpaeoniflorin/paeoniflorin_	0.101 ± 0.002	0.101 ± 0.002	0.101 ± 0.003	0.101 ± 0.002
*f*_pentagalloylglucose/paeoniflorin_	0.0417 ± 0.0010	0.0411 ± 0.0012	0.0404 ± 0.0010	0.0411 ± 0.0011
*f*_benzoic acid/paeoniflorin_	0.190 ± 0.005	0.191 ± 0.006	0.212 ± 0.004	0.197 ± 0.005
*f*_benzoylpaeoniflorin/paeoniflorin_	0.607 ± 0.005	0.609 ± 0.008	0.595 ± 0.018	0.604 ± 0.010
*f*_paeonol/paeoniflorin_	0.0202 ± 0.0005	0.0202 ± 0.0006	0.0201 ± 0.0003	0.0202 ± 0.0005

**Table 6:** The effects of column temperatures, flow rates, columns, and instruments on the relative correction factors.

Relative correction factor	Temp-25^*a*^	Temp-26^*a*^	Temp-28^*a*^	Temp-29^*a*^	Flow-0.90^*b*^	Flow-0.95^*b*^	Flow-1.02^*b*^	Column-YMC^*c*^	Column-BEH^*d*^	Instrument-A^*e*^	Instrument-U^*f*^
*f*_gallic acid/paeoniflorin_	0.0361	0.0365	0.0349	0.0350	0.0357	0.0359	0.0354	0.0329	0.0323	0.0359	0.0364
*f*_oxypaeoniflorin/paeoniflorin_	0.0610	0.0622	0.0620	0.0625	0.0614	0.0625	0.0618	0.0564	0.0548	0.0598	0.0591
*f*_catechin/paeoniflorin_	0.154	0.157	0.154	0.153	0.147	0.156	0.150	0.145	0.143	0.155	0.146
*f*_albiflorin/paeoniflorin_	1.10	1.10	1.12	1.14	1.14	1.13	1.09	1.05	1.04	1.15	1.16
*f*_ethyl gallate/paeoniflorin_	0.0337	0.0334	0.0323	0.0319	0.0335	0.0332	0.0328	0.0310	0.0319	0.0323	0.0330
*f*_galloylpaeoniflorin/paeoniflorin_	0.104	0.103	0.100	0.0995	0.102	0.102	0.102	0.104	0.0999	0.102	0.100
*f*_pentagalloylglucose/paeoniflorin_	0.0423	0.0420	0.0409	0.0411	0.0423	0.0419	0.0417	0.0421	0.0435	0.0423	0.0439
*f*_benzoic acid/paeoniflorin_	0.203	0.199	0.199	0.198	0.200	0.194	0.197	0.190	0.198	0.198	0.194
*f*_benzoylpaeoniflorin/paeoniflorin_	0.639	0.634	0.603	0.608	0.608	0.615	0.624	0.601	0.601	0.615	0.619
*f*_paeonol/paeoniflorin_	0.0212	0.0212	0.0203	0.0210	0.0206	0.0207	0.0211	0.0201	0.0207	0.0206	0.0208

^a^The column temperature was changed to 25, 26, 28, or 29°C. ^*b*^The flow rate was changed to 0.90, 0.95, or 1.02 mL/min. ^*c*^The column was changed to a YMC-Pack Pro C18 column (5 *μ*m, 250 × 4.6 mm) and the flow rate was changed to 0.95 mL/min. ^*d*^The column was changed to a Waters BEH C18 column (5 *μ*m, 250 × 4.6 mm) and the flow rate was changed to 0.95 mL/min. ^*e*^The instrument was changed to an Agilent 1260 system (Agilent Technologies Inc., Waldbornn, Germany). ^*f*^The instrument was changed to a Dionex UltiMate 3000 system (Thermo Scientific Inc., Germering, Germany).

**Table 7:** The effects of biased detection wavelengths on the relative correction factors.

Relative correction factor	−2 nm biased	−1 nm biased	+1 nm biased	+2 nm biased
*f*_gallic acid/paeoniflorin_	0.0338 (272/272)^*a*^	0.0346 (273/273)	0.0354 (275/275)	0.0352 (276/276)
*f*_oxypaeoniflorin/paeoniflorin_	0.0610 (255/272)	0.0620 (256/273)	0.0608 (258/275)	0.0603 (259/276)
*f*_catechin/paeoniflorin_	0.159 (272/272)	0.161 (273/273)	0.147 (275/275)	0.141 (276/276)
*f*_albiflorin/paeoniflorin_	1.09 (272/272)	1.09 (273/273)	1.09 (275/275)	1.09 (276/276)
*f*_ethyl gallate/paeoniflorin_	0.0320 (272/272)	0.0327 (273/273)	0.0333 (275/275)	0.0331 (276/276)
*f*_galloylpaeoniflorin/paeoniflorin_	0.101 (272/272)	0.101 (273/273)	0.101 (275/275)	0.0995 (276/276)
*f*_pentagalloylglucose/paeoniflorin_	0.0425 (272/272)	0.0423 (273/273)	0.0409 (275/275)	0.0395 (276/276)
*f*_benzoic acid/paeoniflorin_	0.188 (272/272)	0.189 (273/273)	0.193 (275/275)	0.197 (276/276)
*f*_benzoylpaeoniflorin/paeoniflorin_	0.610 (272/272)	0.610 (273/273)	0.621 (275/275)	0.634 (276/276)
*f*_paeonol/paeoniflorin_	0.0203 (272/272)	0.0204 (273/273)	0.0203 (275/275)	0.0201 (276/276)

^a^The biased detection wavelengths (nm) are annotated in the parentheses as (the wavelength of analyte/the wavelength of paeoniflorin).

**Table 8:** The relative retention times (RRTs) of the components in different columns and instruments.

Relative correction factor	Column-Luna^*a*^	Column-YMC^*b*^	Column-BEH^*c*^	Instrument-A^*d*^	Instrument-U^*e*^
RRT_gallic acid/paeoniflorin_	0.14	0.14	0.14	0.15	0.16
RRT_oxypaeoniflorin/paeoniflorin_	0.65	0.62	0.34	0.62	0.69
RRT_catechin/paeoniflorin_	0.69	0.67	0.37	0.67	0.73
RRT_albiflorin/paeoniflorin_	0.92	0.92	0.78	0.92	0.93
RRT_ethyl gallate/paeoniflorin_	1.04	1.02	0.93	1.04	1.05
RRT_galloylpaeoniflorin/paeoniflorin_	1.21	1.23	1.44	1.19	1.21
RRT_pentagalloylglucose/paeoniflorin_	1.26	1.28	1.55	1.25	1.25
RRT_benzoic acid/paeoniflorin_	1.27	1.24	1.26	1.27	1.28
RRT_benzoylpaeoniflorin/paeoniflorin_	1.37	1.42	1.98	1.37	1.34
RRT_paeonol/paeoniflorin_	1.48	1.51	1.99	1.50	1.46

^a^The Phenomenex Luna C18 column was used on the Waters HPLC system. ^*b*^The YMC-Pack Pro C18 column was used on the Waters HPLC system. ^*c*^The Waters BEH C18 column was used on the Waters HPLC system. ^*d*^The instrument was the Agilent 1260 system (Agilent Technologies Inc., Waldbornn, Germany) and the column was the Phenomenex Luna C18. ^*e*^The instrument was the Dionex UltiMate 3000 system (Thermo Scientific Inc., Germering, Germany) and the column was the Phenomenex Luna C18.

**Table 9:** Comparison of the concentrations (*μ*g/mL) of the ten components determined by the external standard method (ESM) and the quantitative analysis of multicomponent with a single-marker (QAMS) method.

Component	Quantitative method/RE^*a*^	RPLP from Pan'an	RPLP from Dongyang	RPLP from Baishan	RPLP from Hangzhou	PRA from Zhangzhongjing Pharmacy	PRA from Renshoutang	PRA from Qixiangge	PRA from Henan Herbal Pharmacy	RPLP from Pan'an^*b*^	RPLP from Dongyang^*b*^	RPLP from Baishan^*b*^	RPLP from Hangzhou^*b*^
Gallic acid	ESM	25.64	25.79	31.29	51.13	149.3	137.3	113.5	168.1	78.6	119.3	111.8	386.0
	QAMS	25.06	25.09	30.68	50.54	150.0	138.5	113.8	168.7	79.91	122.4	113.3	387.4
	RE (%)	−2.3	−2.7	−1.9	−1.2	0.5	0.8	0.2	0.4	1.6	2.6	1.3	0.4

Oxypaeoniflorin	ESM	120.4	143.2	99.61	99.60	78.73	70.71	77.87	78.13	119.7	129.4	79.30	106.2
	QAMS	119.8	142.9	97.64	97.20	75.81	67.75	74.91	75.07	120.6	131.5	77.21	103.8
	RE (%)	−0.5	−0.2	−2.0	−2.4	−3.7	−4.2	−3.8	−3.9	0.7	1.6	−2.6	−2.2

Catechin	ESM	276.2	607.1	61.22	65.54	137.9	118.5	123.1	152.2	/^*c*^	52.10	/	/
	QAMS	286.8	631.0	60.92	65.14	140.8	121.2	125.4	155.6	/	52.51	/	/
	RE (%)	3.8	3.9	−0.5	−0.6	2.2	2.2	1.9	2.2	/	0.8	/	/

Albiflorin	ESM	6183	7913	353.7	2950	1092	908.7	847.4	741.2	4673	4387	234.9	3830
	QAMS	6251	7966	347.2	2953	1090	909.7	844.1	735.9	4782	4511	229.5	3829
	RE (%)	1.1	0.7	−1.8	0.1	−0.1	0.1	−0.4	−0.7	2.3	2.8	−2.3	0.0

Ethyl gallate	ESM	/	/	63.09	70.59	/	/	/	/	/	/	/	/
	QAMS	/	/	61.53	68.91	/	/	/	/	/	/	/	/
	RE (%)	/	/	−2.5	−2.4	/	/	/	/	/	/	/	/

Galloylpaeoniflorin	ESM	154.3	131.3	98.35	131.7	158.8	129.5	133.8	165.8	75.69	66.02	78.02	181.2
	QAMS	154.9	130.3	96.33	130.3	158.8	129.0	132.9	165.8	74.06	64.18	75.96	181.0
	RE (%)	0.4	−0.7	−2.1	−1.1	0.0	−0.4	−0.7	0.0	−2.2	−2.8	−2.6	−0.1

Pentagalloylglucose	ESM	437.7	460.6	251.4	365.0	603.2	404.0	407.2	553.8	/	/	/	/
	QAMS	444.9	465.9	255.8	368.4	609.0	410.6	412.0	558.5	/	/	/	/
	RE (%)	1.7	1.2	1.7	0.9	1.0	1.6	1.2	0.8	/	/	/	/

Benzoic acid	ESM	338.6	572.8	112.0	111.6	/	/	/	/	1245	1231	765.3	658.1
	QAMS	338.9	573.0	109.3	108.5	/	/	/	/	1271	1264	774.4	655.2
	RE (%)	0.1	0.0	−2.3	−2.8	/	/	/	/	2.1	2.6	1.2	−0.4

Benzoylpaeoniflorin	ESM	335.0	368.7	268.8	274.1	149.3	118.8	150.4	165.8	102.3	154.3	110.1	129.7
	QAMS	339.1	372.3	269.4	273.6	145.8	114.8	146.9	162.5	99.00	154.4	106.5	125.0
	RE (%)	1.2	1.0	0.2	−0.2	−2.4	−3.4	−2.3	−2.0	−3.2	0.1	−3.3	−3.7

Paeonol	ESM	15.68	17.64	18.42	/	/	/	/	/	/	/	/	/
	QAMS	15.04	16.96	17.77	/	/	/	/	/	/	/	/	/
	RE (%)	−4.1	−3.8	−3.5	/	/	/	/	/	/	/	/	/

^a^Relative error (RE), calculated as (*C*_QAMS_ − *C*_ESM_)/*C*_ESM_ × 100%. ^*b*^Water was used instead of 70% (v/v) ethanol for the extraction of these batches. ^*c*^“/” means the content is below the LOD.

## Data Availability

All the chromatographic data used to support the findings of this study are available from the corresponding author upon request.
